# Phylogenetic and Phylodynamic Analyses of Soybean Mosaic Virus Using 305 Coat Protein Gene Sequences

**DOI:** 10.3390/plants11233256

**Published:** 2022-11-27

**Authors:** Hoseong Choi, Yeonhwa Jo, Hyunjung Chung, Soo Yeon Choi, Sang-Min Kim, Jin-Sung Hong, Bong Choon Lee, Won Kyong Cho

**Affiliations:** 1 Plant Genomics and Breeding Institute, Seoul National University, Seoul 08826, Republic of Korea; 2 College of Biotechnology and Bioengineering, Sungkyunkwan University, Suwon 16419, Republic of Korea; 3 Crop Foundation Division, National Institute of Crop Science, Rural Development Administration, Wanju 55365, Republic of Korea; 4 Department of Applied Biology, Kangwon National University, Chuncheon 24341, Republic of Korea

**Keywords:** coat protein, haplotype, population, soybean mosaic virus

## Abstract

*Soybean mosaic virus* (SMV) of the family *Potyviridae* is the most devastating virus that infects soybean plants. In this study, we obtained 83 SMV coat protein (CP) sequences from seven provinces in Korea using RT-PCR and Sanger sequencing. Phylogenetic and haplotype analyses revealed eight groups of 83 SMV isolates and a network of 50 SMV haplotypes in Korea. The phylogenetic tree using 305 SMV CP sequences available worldwide revealed 12 clades that were further divided into two groups according to the plant hosts. Recombination rarely occurred in the CP sequences, while negative selection was dominant in the SMV CP sequences. Genetic diversity analyses revealed that plant species had a greater impact on the genetic diversity of SMV CP sequences than geographical origin or location. SMV isolates identified from *Pinellia* species in China showed the highest genetic diversity. Phylodynamic analysis showed that the SMV isolates between the two *Pinellia* species diverged in the year 1248. Since the divergence of the first SMV isolate from *Glycine max* in 1486, major clades for SMV isolates infecting *Glycine* species seem to have diverged from 1791 to 1886. Taken together, we provide a comprehensive overview of the genetic diversity and divergence of SMV CP sequences.

## 1. Introduction

*Soybean mosaic virus* (SMV) is a plant virus belonging to the genus *Potyvirus* in the family *Potyviridae*. SMV is usually transmitted by aphids and seeds [1]. SMV mostly infects plants in the Fabaceae family [2]. In particular, when cultivated soybeans (*Glycine max* (L.) Merr.) are infected with SMV, it leads to severe viral symptoms such as mosaic, mottling, leaf distortion, and curling.

Soybeans are a useful source of dietary protein and oil in humans in most Asian countries and are also used as animal feed in Western countries [3]. The cultivated soybean was domesticated from the wild soybean (*Glycine Soja Sieb. & Zucc.*) in East Asia 6000–9000 years ago [4]. Historically, soybean domestication has been reported in northeastern China, and several previous studies using molecular genetic markers suggest that the Yellow River basin might be the origin of soybean domestication [4]. In Korea, soybeans have been widely used in many soybean-based foods, such as soy sauce, soybean paste, and soybean curd, since approximately 4000 years ago [5].

Owing to the importance of soybean cultivation in Asian countries, including Korea and China, studies on pathogens associated with soybeans have been actively conducted. For example, a recent study used RT-PCR and RNA sequencing to conduct a systematic analysis of viruses infecting soybeans in Korea [6]. This study demonstrated that SMV was the most prominent among the 10 identified RNA viruses infecting soybeans.

The SMV genome consists of a single-stranded positive RNA that possesses two open reading frames (ORFs) encoding polyprotein and Pretty Interesting *Potyviridae* ORF (PIPO). After the first complete genome sequence of SMV strain N was reported, the genome sequences of several SMV strains were reported in the United States of America (USA) [7,8]. Subsequently, several SMV genome sequences have been identified in diverse countries. For instance, in Korea, a previous study reported the complete genome sequences of 30 SMV isolates/strains identified in *G. max* and *G. soja* [9]. In China, the complete genome sequences of 18 SMV isolates have been determined [10]. Furthermore, in Iran, several partial nucleotide sequences of the SMV polyprotein covering P1, P3, 6K1, cytoplasmic inclusion (CI), and coat protein (CP) of 26 SMV isolates from *G. max* have been reported [11].

To date, most SMV gene sequences have been obtained from *Glycine* species; however, some SMV sequences have been obtained from other plant species. For instance, the complete genome sequence of SMV isolate P-1 and the CP sequences of several SMV isolates identified from *Pinellia ternata* have been reported [12,13]. Moreover, the genome sequences of SMV isolates from *Passiflora* species in Ecuador and Colombia have been reported [14,15].

CP plays an important role in the protection of genetic material inside viruses. CP has many additional activities involved in the viral life cycle and defense response of host plants against viral infections. Moreover, viral CP sequences have been implicated in a wide range of applications, such as virus diagnosis, viral population study, genetic diversity, and phylogenetic analysis. For instance, genetic diversity and phylogenetic analyses using CP sequences have been studied for several plant viruses, including tomato chlorosis virus [16], Southern tomato virus [17], potato virus Y [18], Arabis mosaic virus [19], and ornithogalum mosaic virus [20].

In this study, we amplified and sequenced 83 SMV CP sequences collected from soybean plants displaying severe disease symptoms from seven provinces in Korea, and CP sequences were used for phylogenetic, genetic diversity, and haplotype analyses. Based on 83 SMV sequences, the genetic diversity of SMV CP sequences in Korea and their genetic relationships according to haplotype were revealed. Furthermore, by combining all available SMV CP sequences from GenBank, we obtained 305 SMV CP sequences that were used for phylogenetic, haplotype, genetic diversity, and phylodynamic analyses. These comprehensive analyses revealed the genetic diversity of SMV CP sequences in Korea and their genetic relationships according to haplotype. Finally, the most important factor (geographical region or plant host) for the genetic diversity of SMV CP sequences was elucidated in our study.

## 2. Results

### 2.1. Genetic Diversity Analysis of the SMV CP Sequences Obtained in This Work

**Figure 1:**
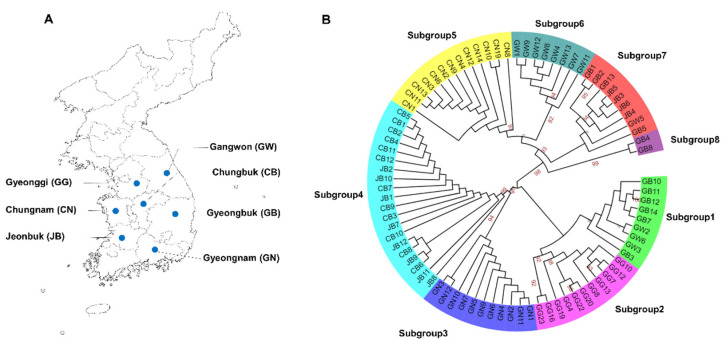
Geographical locations of seven provinces and phylogenetic relationship of 83 SMV CP sequences. (**A**) Geographical locations of seven provinces in Korea in which samples were collected. (**B**) Maximum likelihood phylogenetic tree of 83 SMV CP nucleotide sequences. The eight identified subgroups are indicated by different colors. Bootstrap values > 75% were indicated.

A total of 150 soybean samples showing severe viral disease symptoms were collected from seven different provinces in Korea. After extracting the total RNA from the collected leaf samples, we conducted RT-PCR using a primer pair amplifying the sequence covering the SMV CP. The individual amplified PCR product was cloned, followed by Sanger sequencing to determine the sequences. Finally, we obtained 83 SMV CP sequences that were 690 base pairs (bp) in length (Table S1). We named the sequences according to the name of the collected province, Chungbuk (CB), Chungnam (CN), Gyeongbuk (GB), Gyeonggi (GG), Gyeongnam (GN), Gangwon (GW), and Jeonbuk (JB) (Figure 1A).

The 83 SMV CP sequences were aligned and used for the nucleotide diversity analysis. The number of sequences obtained ranged from 11 (GG and GN) to 13 (CN) (Table 1). The number of segregating sites differed dramatically across the seven provinces (populations). For example, the number of segregating sites in CB was only three, whereas 101 were detected in GB. The number of haplotypes varied from 3 (GN) to 12 (GB). Haplotype diversity was very high in GB (1), while CB showed the lowest haplotype diversity (0.45455). The average number of genetic differences was low in four provinces, namely CB, CN, GG, and GN, whereas the average number of genetic differences was very high in GB, GW, and JB. The nucleotide diversity in the seven provinces ranged from 0.00057 (CB) to 0.04395 (GB).

### 2.2. Phylogenetic Analysis of the SMV CP Sequences Obtained in This Work

**Figure 2:**
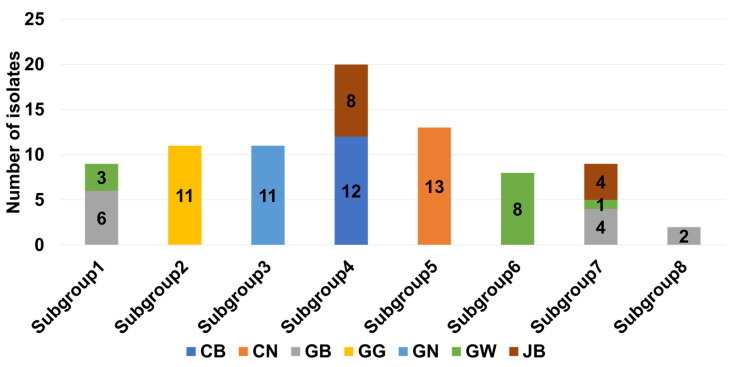
The composition of 83 SMV CP sequences according to the identified province in each subgroup based on the phylogenetic tree in Figure 1B.

We aligned 83 SMV CP nucleotide sequences and generated a maximum likelihood phylogenetic tree, which revealed eight subgroups (Figure 1B). In general, these eight subgroups can be divided into two groups. We analyzed the origin of the SMV sequences according to the identified province (population) in each subgroup in detail (Figure 2). Five of eight subgroups, namely subgroups 2, 3, 5, 6, and 8, contain SMV CP sequences identified from the same province. In contrast, subgroups 1, 4, and 7 consists of SMV sequences identified from the different provinces.

### 2.3. Haplotype Analysis of the SMV CP Sequences Obtained in This Work

**Figure 3:**
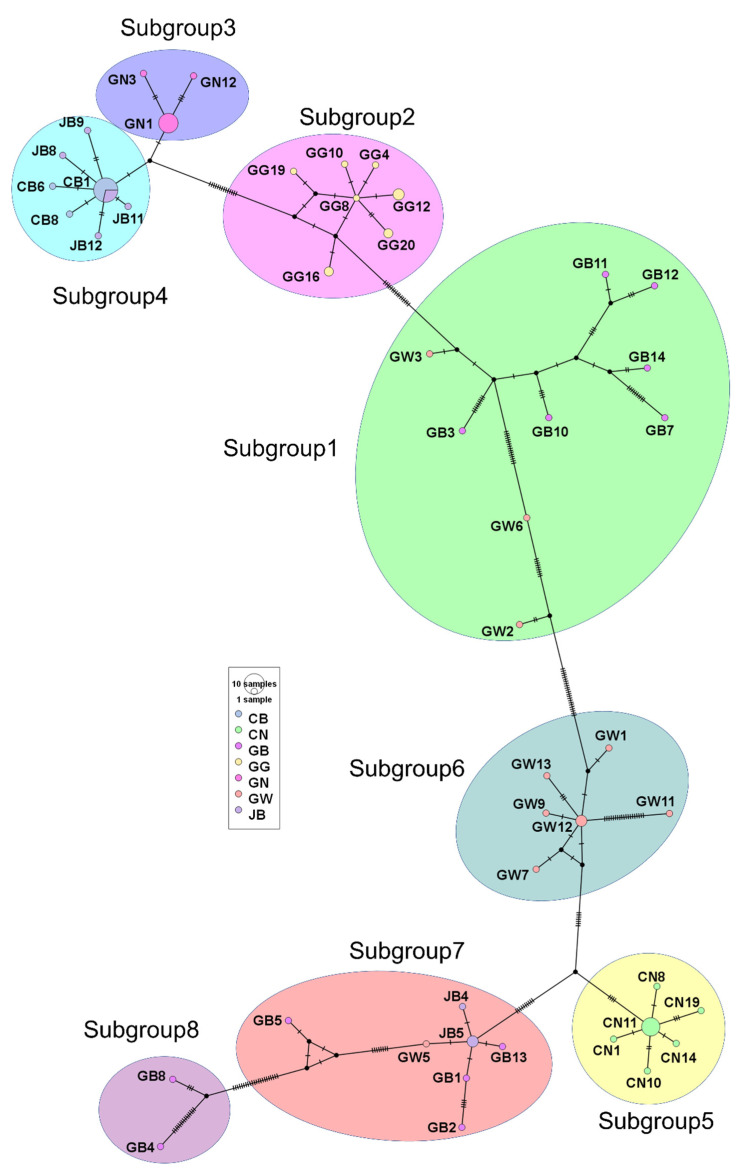
Haplotype network of 83 SMV sequences based on CP sequences. Minimum spanning network was constructed using PopART. The defined eight subgroups were indicated by different colors. Haplotype colors correspond to different provinces (population). The sample sizes in the given haplotype are indicated by the circle size. Each mutation is marked by hatch marks.

Haplotype network is a useful method for determining the relationships between different virus populations. A minimum spanning network of 83 SMV sequences was generated. The haplotype network also revealed eight subgroups defined by 50 haplotypes (Figure 3). The high-frequency haplotypes were CB1 (14 sequences) in subgroup 4, GN1 (nine sequences) in subgroup 3, and CN11 (eight sequences) in subgroup 5 (Table S2). Three haplotypes (GW12, JB5, and GG12) contained three sequences, while two haplotypes (GG16 and GG20) contained two. High-frequency haplotypes with multiple connections might be older. For example, CB1 was connected with seven different haplotypes (Figure 3). In contrast, two haplotypes (GB4 and GB8) in subgroup 8 may be newer. Moreover, two haplotypes (GN3 and GN12) in subgroup 3 may have recently evolved from GN1. The haplotypes in subgroups 1 and 7 had a broader distribution, indicating a high degree of genetic diversity among haplotypes. However, the haplotypes in subgroups 4 and 5 had a narrower distribution, suggesting a low degree of genetic diversity among haplotypes. The hatch marks in the network indicate the number of mutations between haplotypes. For example, three haplotypes (CN1, CN8, and CN14) in subgroup 5 labeled by a single bar indicated that the three haplotypes had recently evolved from CN11 with a single mutation. In contrast, GW11 in subgroup 6 had 19 hatch marks (mutations), which may have evolved from GW12 with a high level of mutations.

### 2.4. Phylogenetic Analysis of SMV

**Figure 4:**
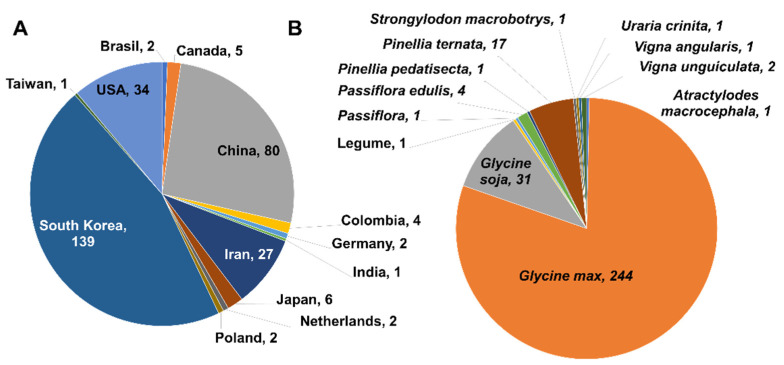
Information of 305 SMV CP sequences used for phylogenetic analysis. Pie chart displays the distribution of 305 SMV sequences according to countries (**A**) and plant host (**B**).

Next, we combined 222 SMV CP sequences from GenBank and 83 CP sequences from this study, resulting in 305 SMV CP sequences (Table S3). A total of 305 SMV sequences were obtained from 13 countries (Figure 4A) and were identified from 11 plant species (Figure 4B). The majority of SMV sequences were derived from South Korea (139 sequences), followed by China (80 sequences), the USA (34 sequences), and Iran (27 sequences). Most SMV sequences were identified in *G. max* (244 sequences), followed by *G. soja* (31 sequences), and *Pinellia ternata* (17 sequences).

**Figure 5:**
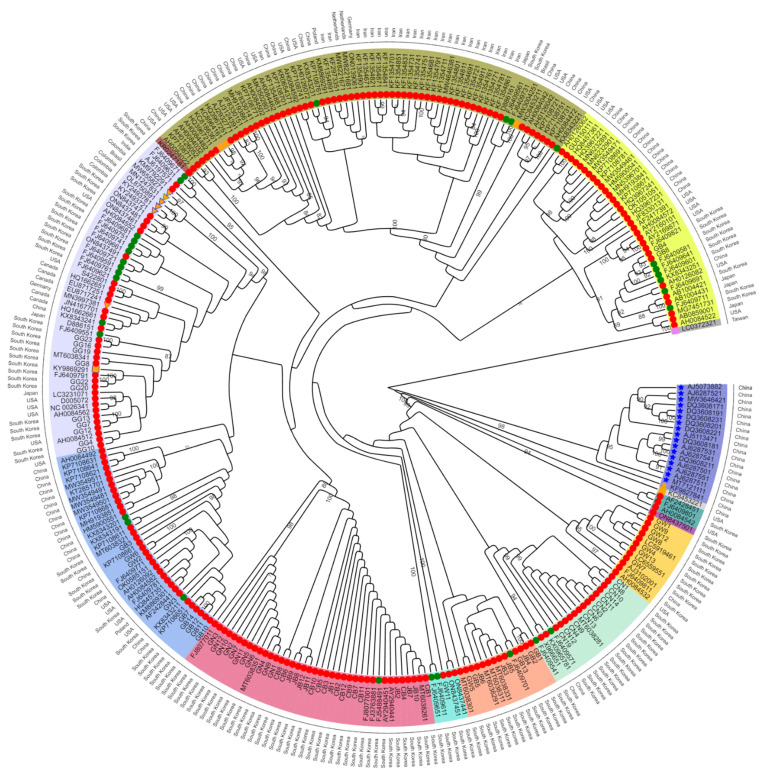
The phylogenetic relationship of 305 SMV sequences. Maximum likelihood phylogenetic tree of 305 SMV sequences based on CP sequences. The rectangular cladogram of phylogenetic tree can be found in Figure S1. Different background leaf colors represent the 12 recognized clades. Bootstrap values > 75% are indicated. Plant species are indicated as follows. *Atractylodes macrocephala* (rectangle, orange: orange), *Glycine max* (circle, red), *Glycine soja* (circle, green), legume (triangle, orange: red), *Passiflora* (triangle, orange: green), *Passiflora edulis* (triangle, orange: blue), *Pinellia pedatisecta* (star, orange), *Pinellia ternate* (star, yellow), *Strongylodon macrobotrys* (rectangle, orange), *Uraria crinite* (rectangle, violet), *Vigna angularis* (rectangle, orange: blue), and *Vigna unguiculata* (rectangle, orange: violet).

**Figure 6:**
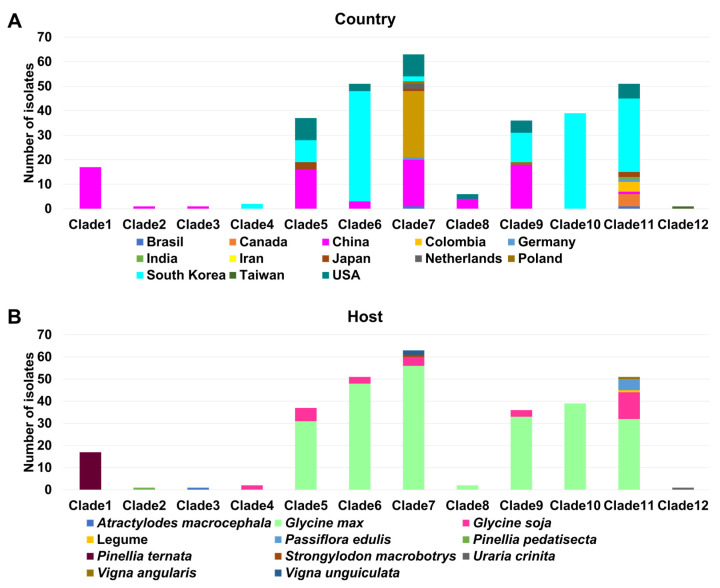
Composition of 305 SMV sequences in each clade. The composition of SMV sequences in each clade according to the country (**A**) and plant host (**B**) was examined.

To reveal the phylogenetic relationship of the 305 SMV sequences, we generated a maximum likelihood phylogenetic tree based on the 305 SMV CP nucleotide sequences, revealing 12 clades (Table S3, Figure 5, and Figure S1). The number of SMV sequences in each clade ranged from 63 sequences (clade 7) to one sequence (clade 2, clade 3, and clade 12). We examined the composition of the SMV sequences in each clade according to the identified country (Figure 6A). Among the clades possessing more than 10 sequences, clade 1 (17 sequences) from China and clade 10 (39 sequences) from South Korea were each derived from a single country. Based on the identified plant hosts, most of the SMV sequences were derived from *G. max* (Figure 6B). All 17 sequences in clade 1 were exclusively derived from *P. ternata* while clade 10 consisted of 39 *G. max* sequences. Clade 11 consisted of five plant hosts.

### 2.5. Genetic Diversity of SMV

Recombination contributes to the genetic diversity of RNA viruses. To identify recombination events within the SMV CP sequences, the aligned SMV CP sequences were subjected to network analysis using the SplitsTree4 program. The network tree displayed several reticulated nodes, suggesting possible recombination events within SMV CP sequences (Figure S2). Using the RDP5 program, we examined recombination events. We only identified a single recombinant (MT603832.1 isolate GYBU-p) from *G. max* in South Korea (Table S4). Moreover, we identified 16 major parental sequences and 107 minor parental sequences associated with the recombinant (isolate GYBU-p).

We examined the genetic diversity of 305 SMV CP sequences. The CP coding sequences were aligned and subjected to genetic diversity analysis using DNASP6. The genetic diversity of the 305 SMV CP sequences was S = 357, Eta = 481, Pi = 0.06276, *θw* = 0.08426, and Tajima’s *D* = –1.392068 (Table S5). We grouped CP sequences according to the identified country and plant host. We then examined the genetic diversity of SMV CP sequences in the four major countries and plant hosts (Table S5). The number of segregating sites (S) for SMV CP in the four major countries ranged from 269 (China) to 24 (Iran). Moreover, the total number of mutations in SMV CP in the four countries ranged from 322 (China) to 24 (Iran). Among the four major plant hosts, *G. max* displayed the highest number of segregating sites (S = 277) and total number of mutations (Eta = 326). In contrast, *Passiflora* species showed the lowest number of segregating sites (S = 20) and total number of mutations (Eta = 20).

**Figure 7:**
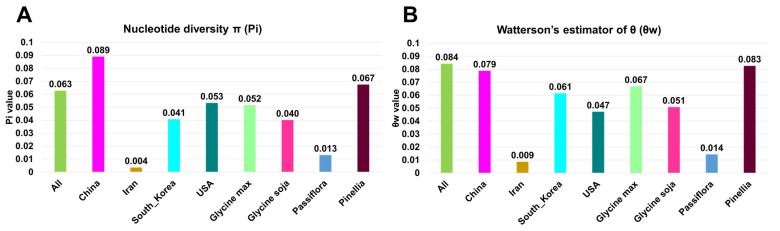
Genetic diversity of SMV CP sequences. Nucleotide diversity (Pi) (**A**) and the average number of segregating sites (*θw*) (**B**) of 305 SMV sequences according to country and plant host. “All” indicates all 305 SMV sequences. Four major countries and four major host plants were examined.

We compared the nucleotide diversity π (Pi) in eight different groups grouped by country or plant host (Figure 7A). Compared to 305 SMV CP sequences defined by ‘all’ (Pi = 0.063), the Pi values in most groups were less than 0.063, except for China (Pi = 0.089) and the *Pineallia* species (Pi = 0.067). Among the four countries, nucleotide diversity was highest in China and lowest in Iran (Pi = 0.004). Similarly, the nucleotide diversity of the *Pinellia* species (Pi = 0.067) was the highest, whereas that of the *Passiflora* species (Pi = 0.013) was the lowest.

Next, we calculated the average number of segregating sites (*θw*) for each group (Figure 7B). The average number of segregating sites in ‘all’ (*θw* = 0.084) was the highest among the nine groups, followed by the *Pinellia* species (*θw* = 0.083), China (*θw* = 0.079), and *G. max* (*θw* = 0.067). Iran (*θw* = 0.009) had the lowest average number of segregating sites, followed by the *Passiflora* species (*θw* = 0.014).

**Figure 8:**
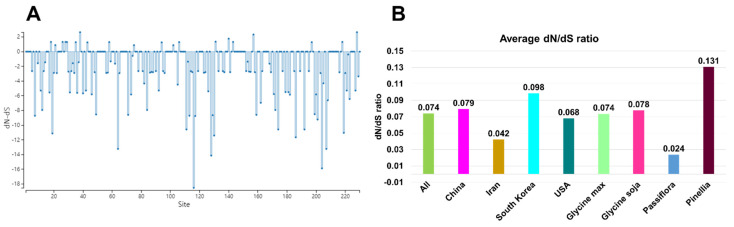
Average dN/dS ratios for 305 SMV CP proteins. (**A**) Distribution of dN/dS ratio for 83 SMV CP proteins from seven different provinces in Korea. (**B**) Average dN/dS ratio for SMV CP proteins grouped in four countries and four plant hosts, respectively. “All” indicates 305 SMV CP proteins.

We analyzed the ratio of non-synonymous to synonymous substitutions (dN/dS) for 305 SMV CP sequences to measure natural selection acting on CP protein-coding genes [21]. Using the single-likelihood ancestor counting (SLAC) method, we identified 0 sites of positive/diversifying selection and 23 sites of negative/purifying selection with *a p-value* of 0.05 set as the cutoff for the 83 SMV CP sequences from seven provinces in Korea. The SLAC site graph showed that most sites in the 83 SMV CP sequences had negative values for the dN/dS ratio (Figure 8A). The average dN/dS ratio for all 305 SMV proteins was 0.074 (Figure 8B). The average dN/dS ratios for the SMV CP in the four major countries ranged from 0.098 (South Korea) to 0.042 (Iran). Among the four major plant hosts, the average dN/dS ratio for SMV CP proteins in the *Passiflora* species (0.024) was the lowest, whereas that in the *Pinellia* species (0.131) was the highest. Overall, the SMV CP proteins showed strong negative selection.

### 2.6. Haplotype Analysis of SMV

**Figure 9:**
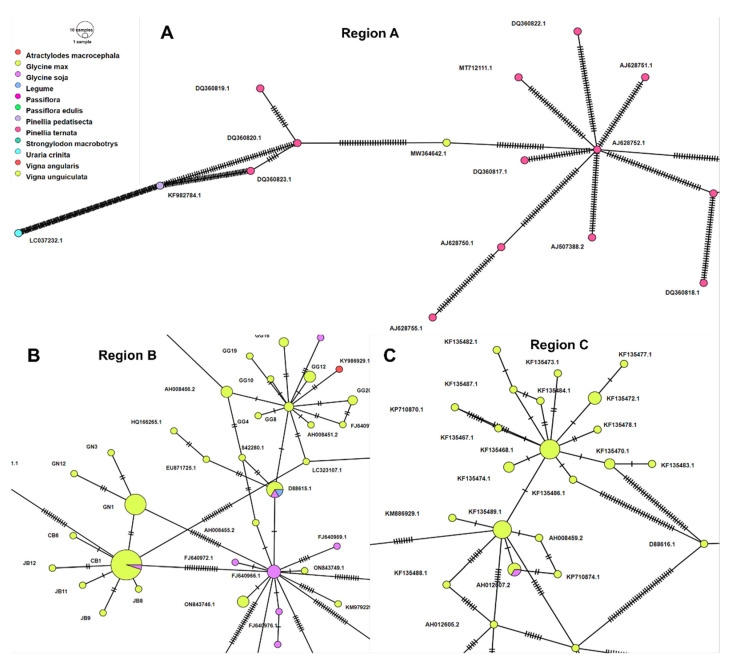
Haplotype network of 305 SMV CP sequences. Minimum spanning networks were implemented in PopART. Three regions, defined as region A (**A**), region B (**B**), and region C (**C**), were magnified from the complete haplotype network shown in Figure S3. Haplotype colors correspond to different plant hosts. The sample sizes in the given haplotype are indicated by the circle size. Each mutation is marked by hatch marks. The original network can be found in Figure S3.

We conducted a haplotype analysis using 305 SMV CP sequences. A total of 198 haplotypes were visualized using a minimum spanning network in the PopART program (Figure S3). The haplotype network showed a relationship between 198 haplotypes, revealing a complex of SMV CP haplotypes with several groups (Figure S3). In general, haplotypes derived from the same plant host preferentially clustered together. Among the identified 198 haplotypes, some haplotypes, such as CB1 (21 sequences), CN11 (10 sequences), and GN1 (10 sequences), represented higher numbers of SMV sequences (Table S6). We magnified three specific regions of the haplotype networks. Haplotypes in region A corresponded to clades 1, 2, and 12 of the maximum likelihood phylogenetic tree (Figure 9A). The haplotypes in region A were mostly derived from two *Pinellia* species and *Uraria crininata*. The hash marks indicate a high degree of mutations between haplotypes in region A. In region B, CB1 was related to nine haplotypes, including GN1 (Figure 9B). Besides the three networks, there were few mutations between CB1 and the other CB1-related haplotypes. Most sequences in haplotype CB1 were derived from *G. max*, with the exception of some sequences from *G. soja*. The haplotype FJ640966.1 from *G. soja*, was connected to 15 haplotypes from *G. max* and *G. soja* (Figure 9B). The haplotype D88615.1 was connected to four haplotypes containing six sequences identified from *G. max*, *G. soja*, and legumes (Figure 9B). In region C, two haplotypes, KF135468.1 and KF135489.1, were connected to many haplotypes (Figure 9C). KF135468.1 (seven sequences) was connected to at least 10 haplotypes, with a low number of mutations among the haplotypes. KF135489.1 (six sequences) was connected to eight haplotypes, and of the connected haplotypes, four (KM886929.1, AH012607.2, AH008459.2, and KF135486.1) showed low levels of mutation.

### 2.7. Phylodynamic Analysis of SMV

We estimated the relative times of divergence for 305 SMV sequences based on the CP sequences and their identified years using the TreeTime program (Table S7). Since the first complete sequence of SMV isolate N (D00507.2) from *G. max* in the USA in 1989, the sequences of many SMV genes have been reported. In 1999, 23 CP sequences of 23 SMV sequences from the USA were reported (Table S3). In Korea, since the first report of SMV gene sequences in 2003, 93 SMV gene sequences were reported in 2016. In Iran, 26 SMV gene sequences from 2004 onwards are currently available. In China, many SMV gene sequences have been made available from 1993 to 2021.

**Figure 10:**
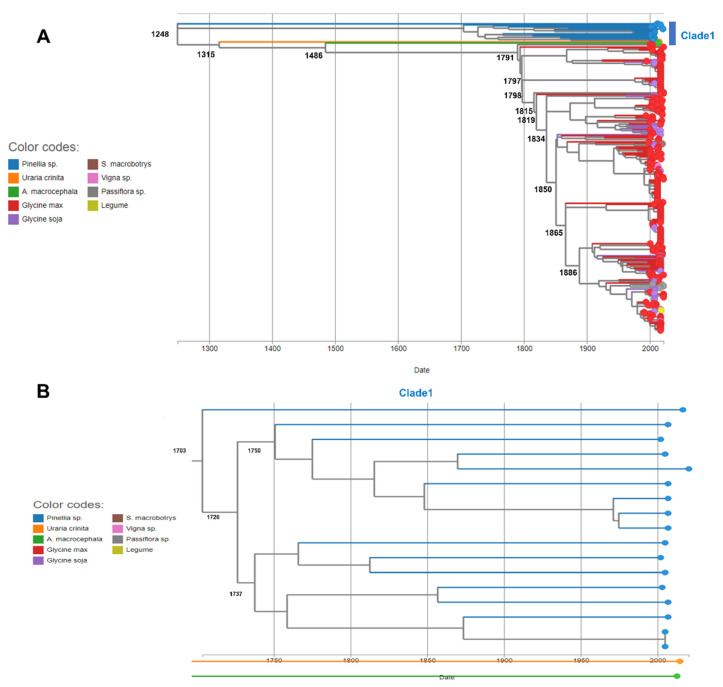
Phylogenomic analyses of 305 SMV CP sequences. (**A**) A maximum likelihood phylogenetic tree with estimated divergence time for 305 SMV CP sequences was created using TreeTime. Individual sequences are indicated by circles and plant hosts are indicated by different colors. Major nodes (branching points) are indicated by the estimated time. (**B**) Time-resolved tree of clade 1.

The maximum likelihood phylogenetic tree shows the estimated relative time of each node (branching points) (Table S7 and Figure 10A). The phylogenetic tree revealed two groups of the 305 SMV sequences. The first group included clades 1 and 2, which were composed of *Pinellia* spp. The second group, which contained 287 SMV sequences (clades 3–12), was derived from *G. max* and other plant hosts. The estimated divergence time between clades 1 and 2 was the year 1248. Clade 2 contained only one SMV isolate, NN (KF982784.1), identified from *Pinellia pedatisecta* in China. Clade 1 consisted of 16 SMV sequences from *P. ternata* and one sequence from *G. max*. The divergence time of SMV sequences in clade 1 ranged from 1703 to 1750 (Figure 10B). Among the SMV sequences in the second group, the SMV isolate Uraria (LC037232.1), identified from *Uraria crinita* in Taiwan in 2014, a member of clade 12, diverged in the year 1315 from other SMV sequences. SMV isolate Am (KC845322.1), which was isolated from *Atractylodes macrocephala* in China in 2012 and is a member of clade 3, diverged from other SMV sequences in 1486.

Three sequences annotated as G6 (AH008454.2, FJ640980.1, and AF242845.1) in clade 6, identified from *G. max*, diverged in 1791. After G6, SMV isolate GR4 (ON843750.1) from Korea diverged in 1797. The estimated divergence times for most clades containing SMV sequences identified from *G. max* and *G. soja* ranged from 1791 to 1886. The regression of root-to-tip genetic distance against sampling time was R^2^ = 0.001, with μ = 2.738e-4.

## 3. Discussion

SMV is regarded as the most important and devastating virus affecting soybeans [22]. Recently, the rapid development of diverse sequencing techniques has facilitated studies of the phylogenetic and genetic diversity of target plant viruses [23]. Here, we amplified and determined 83 SMV CP sequences derived from seven provinces in Korea. Based on the 83 CP sequences, we conducted population, haplotype, and genetic diversity analyses of Korean SMV sequences identified from soybean plants. Prior to this study, we assumed that the genetic diversity of SMV CP sequences from cultivated soybean plants in Korea might be low. Unexpectedly, most SMV CP sequences obtained from individual provinces (populations) showed strong nucleotide diversity. This result is consistent with a previous study, which demonstrated that most plant virus populations show a high degree of genetic variation and diversity [24]. Among the seven different provinces (populations), the genetic diversity of the SMV CP sequences was much higher in GB and GW and lower in CB and GN. This result implies that geographical region is also an important factor in determining the genetic variation of SMV CP sequences.

Both the maximum likelihood phylogenetic tree and haplotype network revealed eight groups of 83 SMV sequences in Korea. SMV sequences from the same province preferentially clustered together, indicating that these SMV isolates might have evolved from the same pre-existing SMV isolate/strain. Some subgroups comprised SMV sequences from different provinces that were geographically connected. For example, GB and GW were grouped together. It seems that insect vectors, such as aphids or seed-borne infections, could contribute to the transmission of SMV between two different regions, as suggested previously [25].

Haplotype network analysis using 50 haplotypes revealed major haplotypes of SMV CP, such as CB1, GN1, and CN11, in Korea that could have emerged long ago. In contrast, GB4 and GB8 in subgroup 8 were newly evolved from pre-existing SMV sequences. Moreover, SMV sequences in subgroups 4 and 5 had narrow genetic diversity with fewer mutations, whereas the SMV sequences in GB4 and GB8 had broader genetic diversity with higher levels of mutations among the sequences. As a result, the construction of a haplotype network using CP sequences provides valuable information associated with Korean SMV populations in the seven geographical regions.

All available SMV CP sequences were collected, resulting in 305 SMV sequences. Most SMV CP sequences were derived from 2 out of 13 countries, namely South Korea and China. *G. max and G. soja* are the major hosts of SMV. Furthermore, diverse plant species, such as *A. macrocephala*, *P. edulis*, *P. pedatisecta*, *P. ternata*, *Stronglylodon macrobtrys*, and *U. crinita*, are also hosts for SMV. Of these, the SMV sequences from the *Pinellia* (Clade 1) and *Uraria* species (Clade 12) were genetically different from other SMV sequences. Using a BLASTN search, we found that the SMV isolate Uraria (LC037232.1), identified from *U. crinita* in Taiwan in 2014, showed strong sequence similarity to the known uraria mosaic virus (UMV) strain OC (LC477217.1) from *P. edulis* in Japan [26]. Thus, this sequence should be reannotated as UMV based on sequence identity according to the demarcation of potyvirus species by the International Committee on Taxonomy of Viruses (ICTV). However, other SMV sequences from the *Pinellia* species belong to the SMV group based on the demarcation of potyvirus species by ICTV [12,13].

A comprehensive phylogenetic analysis identified 12 clades. Both the phylogenetic tree and haplotype network revealed that the plant host is a more important factor than the identified country for grouping the 305 SMV sequences. For example, the SMV sequences from the *Pinellia* species (Clade 1) were genetically different from other SMV sequences in clades 2–11. Of these, the UMV isolate Uraria in clade 12 was used as the outgroup. Compared to previous phylogenetic analyses using 44 SMV genomes [9] and 104 SMV genome sequences [27], we used 305 CP gene sequences in this study. CP and RNA-dependent RNA polymerase or replicase-like sequences have been widely used as molecular markers for viruses. Gene marker-based phylogenetic analysis may provide detailed information on the phylogenetic relationships of the target virus species. However, obtaining the complete genome sequences of the target virus species is difficult. In contrast, the gene marker-based approach makes it much easier to obtain target gene sequences in a restricted time. Similarly, the RT-PCR method used to amplify CP sequences in this study was very effective at resolving the complex of SMV populations on a large scale.

Our recombination analysis revealed only one recombinant event within the 305 SMV CP sequences. This result indicates that recombination rarely occurs within a restricted gene sequence. Natural selection analysis indicated negative selection of SMV CP protein sequences. Our results were consistent with those of previous studies showing strong negative selection of most SMV protein sequences [27]. Several previous studies have shown high genetic recombination rates in SMV genomes [9,10]. However, it is likely that recombination events might not frequently occur in restricted viral genomic regions, such as CP.

Genetic diversity analyses of the SMV CP sequences were conducted based on the plant host and the identified country. Our results suggest that the impact of plant species on the genetic diversity of SMV CP sequences is much stronger than that by geographical locations. Moreover, SMV sequences identified from China showed the highest number of both segregating sites and total number of mutations, although the number of SMV sequences from Korea was higher than that of China. SMV sequences in China were identified from many diverse plant hosts, whereas SMV sequences in Korea were only identified from *G. max* and *G. soja*. Similarly, SMV sequences identified from the *Pinellia* species in China showed the highest number of both segregating sites and total number of mutations compared to other plant hosts, including *G. max* and *G. soja*. Based on these results, we suppose that the expansion of plant hosts might affect the genetic diversity of SMV CP sequences, as previously suggested [28].

Phylodynamic analysis of 305 SMV sequences estimated the relative time of major branching points and the possible origin of SMV based on the identified time and CP sequences. Based on the maximum likelihood phylogenetic tree, we identified two groups within the 305 SMV sequences that were mostly divided by plant hosts. The phylogenetic tree using 305 CP sequences demonstrated that most SMV sequences identified from *G. max* and *G. soja* might originate from the first group composed mostly of SMV sequences identified as *Pinellia* species. Furthermore, we found that the estimated divergence time between SMV isolates infecting *P. ternata* (clade 1) and SMV isolates infecting *P. pedatisecta* (clade 2) occurred in 1248. This result suggests that there could be strong genetic diversity in the SMV CP sequence between two closely related species. Furthermore, we found that two SMV isolates from *U. crinita* and *A. macrocephala* diverged at 1315 and 1486, respectively. The divergence time gap of the SMV sequences between the different plant species was relatively high. All SMV sequences identified in the *Pinellia*, *Uraria*, and *Atractylodes* species were derived from China. Since the divergence of the first SMV isolate from *G. max* in 1486, the major clades for SMV isolates infecting *Glycine* species seem to have diverged from 1791 to 1886. Taken together, our phylodynamic results provide evidence for the origin and evolutionary timeline of the current SMV isolates/strains worldwide.

## 4. Materials and Methods

### 4.1. Collection of Soybean Samples and Total RNA Extraction

In June 2016, we harvested mature leaves from more than 150 soybean plants showing severe viral disease symptoms from seven different provinces in Korea. The harvested leaves were immediately frozen in liquid nitrogen. Leaf samples were ground using a mortar and pestle with liquid nitrogen. Total RNAs were extracted using a RNeasy Plant Mini Kit (Qiagen, Hilden, Germany), according to the manufacturer’s instructions. The total RNA extracted from each sample was used as a template for RT-PCR.

### 4.2. RT-PCR, Cloning, and Sanger-Sequencing

SMV CP-specific primers SMV_8486_F 5′-GCTGGACTTCAATCATGCTG-3′ and SMV_9377_R 5′-AAAGCGACCCGAAATGATAA-3′ were used for RT-PCR. RT-PCR was performed using the SoGent DiaStar One Step RT-PCR Kit (Daejeon, Korea), according to the manufacturer’s instructions. The following reagents were used: 1 µL of template RNA (100 ng), 1 µL of forward primer (10 pmol/µL), 1 µL of reverse primer (10 pmol/µL), 6 µL of 5× Band Doctor, 6 µL of 5× Buffer, 2 µL of enzyme mix, and RNase-free water to a total volume of 30 µL were added to a 0.2 ml PCR tube for RT-PCR. The RT-PCR conditions were as follows: cDNA synthesis was performed at 50 °C for 30 min, initial denaturation at 95 °C for 15 min, denaturation at 95 °C for 20 s, annealing at 55 °C for 40 s, extension at 72 °C for 40 s, 30 repeated denaturation to extension steps, and a final extension at 72 °C for 5 min. The samples were then kept at 4 °C. We examined the amplified PCR products by gel electrophoresis on a 1.5% agarose gel stained with ethidium bromide.

The amplified PCR product on the agarose gel was excised and used for PCR purification using the QIAquick PCR purification kit, according to the manufacturer’s instructions (QIAGEN, Hilden, Germany). The purified PCR product was cloned into a pGEM-T Easy Vector (Promega, Madison, WI, USA). The cloned vector was sequenced in both orientations using Sanger sequencing with universal primers at Macrogen (Seoul, Korea). After trimming primer binding regions, we obtained 83 SMV CP sequences (Table S1).

### 4.3. Collection of SMV-Associated Sequences from GenBank and Sequence Alignment

Using “soybean mosaic virus” as a query in GenBank in NCBI, we downloaded all available SMV-associated sequences (11 August 2022). After combining 83 SMV CP sequences with the downloaded SMV sequences, we aligned all sequences using MAFFT version 7 with the auto option [29]. The aligned sequences were imported into the MEGA7 program, and other unnecessary sequences were manually deleted to obtain only CP coding sequences [30]. In total, 305 SMV CP sequences were obtained. The 305 SMV CP sequences were again aligned using MAFFT version 7 with the auto option. GenBank accession numbers for 305 SMV sequences were used to obtain information for SMV isolates/strains from GenBank.

### 4.4. Generation of Maximum Likelihood Phylogenetic Trees

The aligned 83 SMV CP sequences from this study and 305 SMV CP sequences were subjected to phylogenetic tree construction using IQ-Tree version 2.0 [31]. The generated maximum likelihood phylogenetic tree was imported into Figtree version 1.4.4 (http://tree.bio.ed.ac.uk/software/figtree/). The phylogenetic tree was modified using Evolview version 3 [32].

### 4.5. Recombination Analysis of 305 SMV CP Sequences

We performed recombination analyses for 305 SMV CP sequences using the RDP version Beta 5.28b [33]. A full exploratory recombination scan using seven algorithms (RDP, GENECONV, Bootscan, Maxchi, Chimaera, SiSscan, and 3Seq) implemented in RDP was used for recombination analyses. Of the seven algorithms, recombinants predicted to have a *p*-value less than 0.05 by at least five algorithms were regarded as recombinant.

### 4.6. Haplotype Analysis, Generation of Network Tree, Genetic Diversity Analysis, Calculation of dN/dS, and Phylodynamic Analysis of 305 SMV CP Sequences

The aligned CP sequences were used for the haplotype network using the minimum spanning method implemented in PopART v1.7 [34]. An unrooted network tree was generated using SplitsTree version 5.3.0 [35]. Genetic diversity and dN/dS ratio for 305 CP sequences were analyzed using DnaSP version 6.12.03 [36]. Phylodynamic analysis was conducted using TreeTime version 0.9.4 [37].

## 5. Conclusions

In this study, we obtained 305 SMV CP sequences from seven provinces in Korea using RT-PCR and Sanger sequencing to examine the genetic diversity of SMV sequences in different geographical regions (populations). The phylogenetic tree identified eight groups of 83 SMV sequences. A haplotype analysis revealed a network of 50 SMV haplotypes in Korea. The phylogenetic tree using all available 305 SMV CP sequences revealed 12 clades that were further divided into two groups, which were classified according to different plant hosts. Recombination analysis identified a single recombinant event, indicating that recombination rarely occurred across the 305 CP sequences. Natural selection analysis using the dN/dS ratio revealed that the SMV CP underwent negative selection. Genetic diversity analyses of the SMV CP sequences were conducted based on the plant host and the identified country. Our results suggest that plant species exert a greater impact on the genetic diversity of SMV CP sequences than geographical locations do. Of the examined SMV isolates/strains, isolates identified from the *Pinellia* species in China showed the highest genetic diversity of the SMV CP sequences. Phylodynamic analysis showed that the current SMV sequences infecting cultivated soybeans might have originated from the *Pinellia* species. SMV isolates from two different *Pinellia* species diverged in the year 1227. Since the divergence of the first SMV isolate from *G. max* in 1474, major clades for SMV isolates infecting the *Glycine* species seem to have diverged from 1789 to 1887. Taken together, we provide a comprehensive overview of the origin, divergence, and genetic diversity of SMV isolates/strains using the available SMV CP sequences.

## Figures and Tables

**Table 1:** Genetic diversity analyses of SMV CP sequences between provinces (populations).

Province (Population)	Number of Sequences	Number of Segregating Sites (S)	Average Number of Differences	Nucleotide Diversity π (Pi)
Chungbuk (CB)	12	3	0.5	0.00057
Chungnam (CN)	13	10	1.53846	0.00174
Gyeongbuk (GB)	12	101	38.84848	0.04395
Gyeonggi (GG)	11	10	2.65455	0.003
Gyeongnam (GN)	11	5	0.90909	0.00103
Gangwon (GW)	12	79	25.54545	0.0289
Jeonbuk (JB)	12	58	25.57576	0.02893
All	83	169	33.735	0.03816

## Data Availability

We deposited 83 SMV sequences from this study in the NCBI GenBank database with accession numbers OP389994-OP390076. Detailed information on the 83 SMV sequences can be found in Tables S1 and S3.

## Supplementary Materials

The following supporting information can be downloaded at https://www.mdpi.com/article/10.3390/plants11233256/s1, Table S1: Complete CP sequences of 83 SMV sequences from this study; Table S2: Identical SMV CP sequences in major haplotypes using 83 SMV CP sequences; Table S3: Information of 305 SMV sequences used for phylogenetic analysis; Table S4: Results of recombination analysis for 305 SMV CP sequences; Table S5: Analyses of genetic diversity for 305 SMV CP sequences; Table S6: Identification of SMV CP sequences in major haplotypes using 305 SMV CP sequences; Table S7: Information of SMV sequences, branch length stretch, country, date, host, and estimated relative time of each node (branching points); Figure S1: The rectangular cladogram of maximum likelihood phylogenetic tree of 305 SMV sequences based on CP sequences; Figure S2: Phylogenetic network tree of 305 SMV isolates using CP sequences; Figure S3: Haplotype network of 305 SMV sequences.
