# Supplementary figures and images for: Phylogenetic and Phylodynamic Analyses of Soybean Mosaic Virus Using 305 Coat Protein Gene Sequences
Source: Plants (Basel). 2022 Nov 27;11(23):3256. doi: 10.3390/plants11233256 (PMC9736121; doi:10.3390/plants11233256)

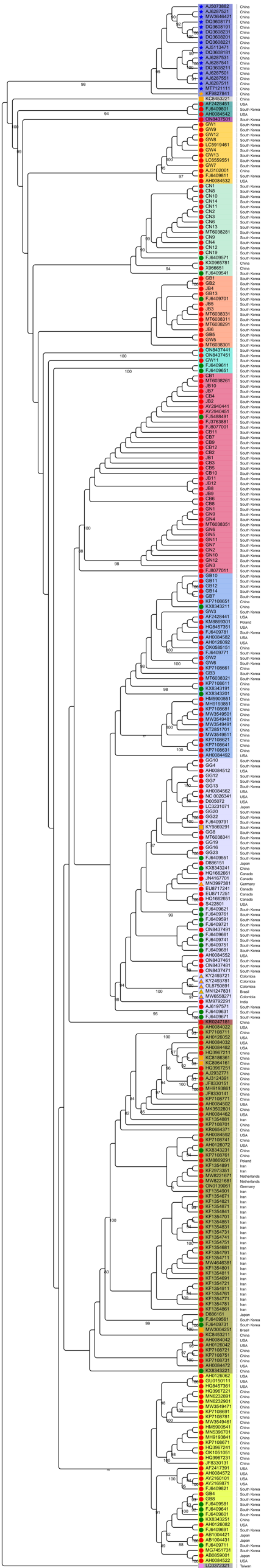

Supplement: Supplementary file 1 [file plants-11-03256-s001.zip › Figure S1.pdf]

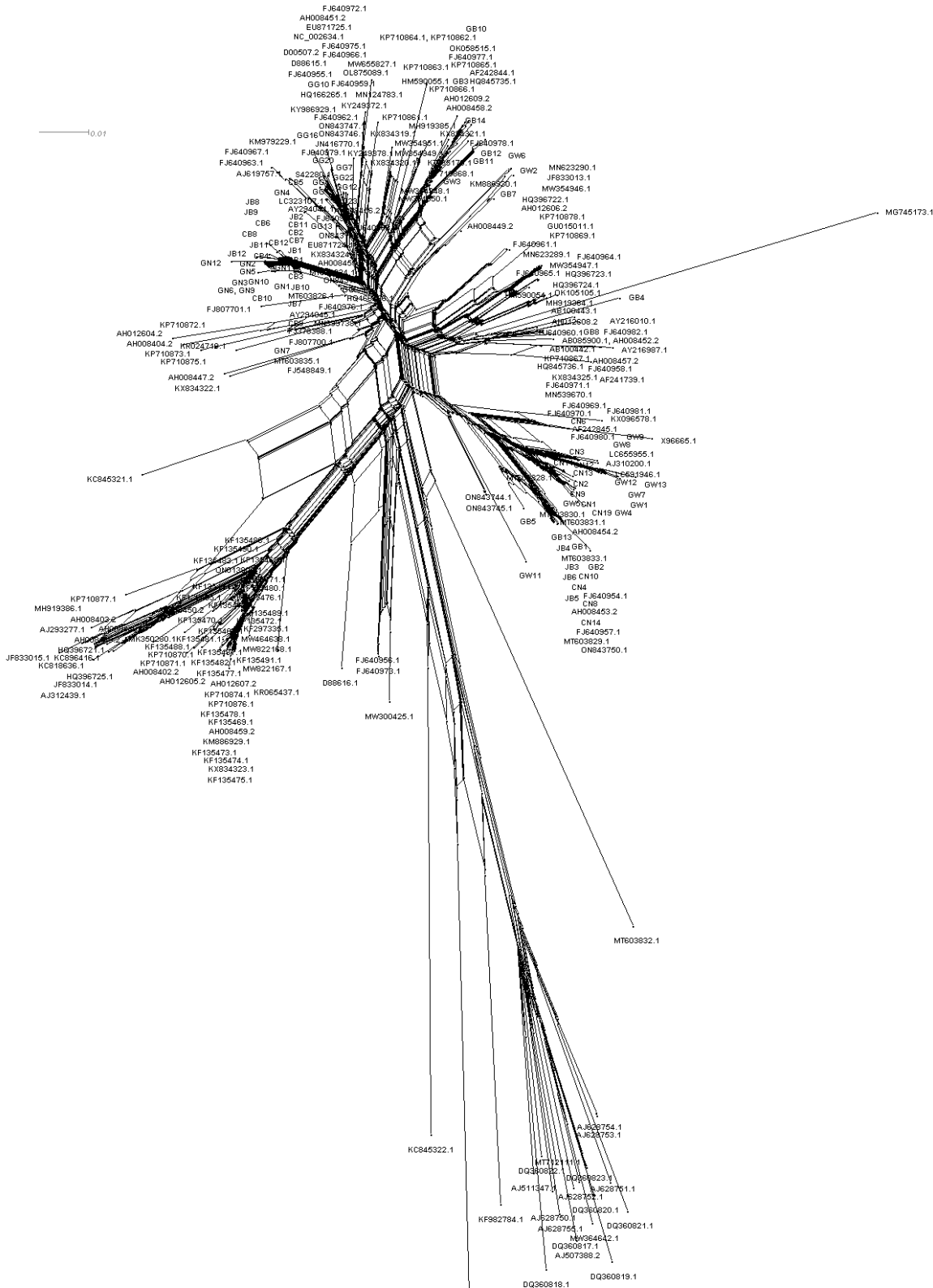

Supplement: Supplementary file 1 [file plants-11-03256-s001.zip › Figure S2.pdf]

Region A

Region C

Region B

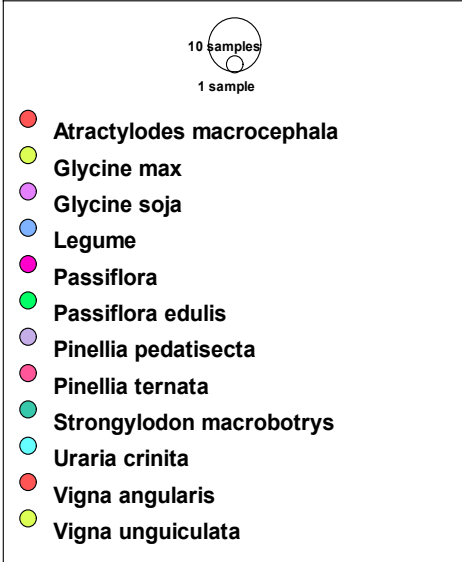

Supplement: Supplementary file 1 [file plants-11-03256-s001.zip › Figure S3.pdf]
